# Abstracts from the Veterinary Emergency and Critical Care Ultrasound Society

**DOI:** 10.1186/s13089-020-00191-6

**Published:** 2020-10-14

## Veccus abstract proceedings Ghent 2020

### Oral presentations

#### Learning curve of novice sonographers to answer binary lung ultrasound questions in companion animals

##### P. Burnotte^1^, S. Boysen^2^, K. Gommeren^1,*^

###### ^1^University of Liège, Faculty of Veterinary Medicine, Liège, Belgium; ^2^University of Calgary, Faculty of Veterinary Medicine, Calgary, Canada

####### **Correspondence:** Kris Gommeren, e-mail: krisjeg@hotmail.com

**Background:** Studies have shown cardiovascular veterinary point-of-care ultrasound (VPOCUS) performed by non-specialists helps differentiate cardiac from respiratory disease, and that a short hands-on training course including interpretation of cineloops improves novice sonographer cardiac VPOCUS accuracy. Studies evaluating sonographer interpretation of LUS cineloops in companion animals are lacking. This study evaluated the accuracy of novice sonographer interpretation of LUS using a binary question approach over a 3-month period. We hypothesized that hands-on VPOCUS training and cineloops interpretation will increase novice sonographer accuracy to answer binary LUS questions.

**Materials and methods:** Twelve interns, with minimal prior ultrasound experience, received a 5-h (1 theory, 4 practical) course on LUS, using a binary question approach. Learner performance to assess LUS findings was assessed prior to (T0), immediately following (T1), and 3 months after training (T3). Between T1 and T3 interns had access to scan clinical patients using VPOCUS, and to record cineloops for review by an experienced VPOCUS clinician.

**Results:** The accurate/inaccurate/unanswered (mean (SD)) responses to binary LUS questions increased from 36.3% (12.8)/15.3% (4.1)/48.4% (13.3) at T0 to 64.6% (9.2)/10.7% (2.8)/24.7% (11.3) at T1 to 85.9% (5.8)/9.8% (3.4)/4.3% (6.2) at T3, respectively. Accuracy for detection of pleural effusion, b-line presence, and b-line quantification was 67.4% (2.6), 74.31% (3.1), and 71.5% (3.1) at T0. Accuracy for the curtain sign, Z lines, lung point, shred sign, double curtain sign, and I-lines was lower at 29.2% (1.4), 22.9% (0.9), 16.7% (0.8), 6.9% (0.4), 4.9% (0.6), and 2.1% (0.4), respectively. At T1, the accuracy of detecting curtain signs, Z lines, double curtain and lung point increased to > 50%, but remained low for I-lines (7.6% (0.9)) and the shred sign (18.1% (1)), with 80% of novices leaving I line and shred sign questions unanswered. At T3 all binary questions were accurately answered > 75% of the time, with > 90% accuracy for double curtain sign (98.6% (0.4)), pleural effusion (93.8% (1.5)), shred sign (93.8% (0.9)), and curtain sign (91% (1.2)).

**Conclusions:** Novice sonographers can rapidly answer most binary questions on LUS with high accuracy following a brief hand on training session and 3 months of clinical practice. Given the difficulty of identifying I-lines and the shred sign, these may be areas requiring greater training. Capture and interpretation of cineloops during clinical practice, with feedback from an experienced VPOCUS operator, appears to improve novice sonographer learner performance rapidly.

#### Evaluation of the utility of point-of-care ultrasound in detecting ureteral obstruction in cats

##### D. Beeston, L. Cole

###### Clinical Science and Services, Royal Veterinary College, Potters Bar, United Kingdom

####### **Correspondence:** Laura Cole, e-mail: lcole3@rvc.ac.uk

**Background:** Ureteral obstruction is a relatively common post-renal cause of azotaemia in cats. Diagnosis can be challenging with many cats exhibiting non-specific clinical signs. The objective of this study was to evaluate the use of point-of-care ultrasound (POCUS) for detection of abnormalities consistent with ureteral obstruction in azotaemic cats.

**Materials and methods:** A retrospective case–control study was conducted of azotaemic cats (serum creatinine > 180 μmol/L) presenting to the emergency room of a referral teaching hospital. Patients were grouped into obstructed and non-obstructed groups based on formal ultrasound by board certified specialist radiologists or supervised residents in-training. Point-of-care ultrasound was performed in all cases by rotating interns or emergency and critical care residents. Data collected included creatinine on presentation and POCUS findings at the hepatorenal and splenorenal site. Diagnosis of ureteral obstruction on POCUS was defined as the presence of one or more of the following: renal asymmetry, pyelectasia, ureteral dilation or visualisation of calculi. Sensitivity, specificity, positive predictive values (PPV) and negative predictive values (NPV) of POCUS for the diagnosis of ureteral obstruction were calculated.

**Results:** One hundred and forty azotaemic cats had both POCUS and formal ultrasound performed during the study period. One hundred cats were included in the obstructed group and 40 cats in the non-obstructed groups. Median creatinine did not significantly differ between the obstructed group (897 μmol/L, range 185–2092) and the non-obstructed group (865 μmol/L, range 199–2616; *P *= 0.135). Point-of-care ultrasound abnormalities were detected in 84/100 obstructed cats and 13/40 non-obstructed cats. The most common POCUS finding in obstructed cats was pyelectasia (*n* = 72), followed by renal asymmetry (*n* = 29), ureteral dilation (*n* = 24), peri-renal fluid (*n* = 17), calculi visualisation (*n* = 11) and bilateral renomegaly (*n* = 8). The most common POCUS finding in non-obstructed cats was peri-renal fluid (*n* = 10) followed by bilateral renomegaly (*n* = 6), and pyelectasia (*n* = 2). Based on our POCUS criteria for the diagnosis of ureteral obstruction, POCUS had a sensitivity of 83.0%, specificity of 82.5%, positive predictive value (PPV) of 92.2% and negative predictive value (NPV) of 66.0% for diagnosis of ureteral obstruction. Documentation of two or more POCUS findings increased the positive predictive value of POCUS to 100%.

**Conclusion:** Point-of-care ultrasound is useful in diagnosing ureteral obstruction in azotaemic cats. One or more POCUS findings, particularly renal asymmetry and pyelectasia, should increase suspicion of ureteral obstruction and prompt further imaging.

### Poster presentations

#### Pilot study on caudal vena cava size and caudal vena cava-to-aortic ratio at the right paralumbar view in healthy Belgian Blue Calves

##### H. Casalta^1^, K. Gommeren^2^, S. Grulke^2^, A. Sartelet^1^, A.-C. Merveille^2^

###### ^1^Clinical Department of Production animals, Faculty of Veterinary Medicine, University of Liège, Belgium; ^2^Clinical Department of Companion animals and Equids, Faculty of Veterinary Medicine, University of Liège, Belgium

####### **Correspondence:** Kris Gommeren, e-mail: krisjeg@hotmail.com

**Background:** Belgian Blue Calves (BBC) are double-muscled beef cattle, representing a valuable investment to the farmer. Hence there is an economic interest to improve treatment of BBCs suffering from surgical or non-surgical digestive diseases, possibly accompanied by severe changes in volume status. Unfortunately, little is known about volume status assessment in bovine medicine. In human and companion animal medicine, ultrasonographic assessment of the inferior/caudal vena cava diameter (CVC_D_) is used as an inexpensive, rapid and noninvasive marker to evaluate intravascular volume status. In companion animals CVC_D_ is often expressed as a ratio to the aortic diameter (Ao_D_) to compensate for variations in body size. To the author’s knowledge the CVC_D_, and the effect of age and body weight on CVC measurements, has never been assessed in BBC calves. The objective of this study was to perform a pilot study on the feasibility to obtain ultrasonographical measurements of the caudal vena cava diameter (CVC_D_) and the aortic diameter (Ao_D_) via the longitudinal right paralumbar (PV) and subxiphoid (SX) view in awake healthy calves, as previously described in dogs. Secondly, we wanted to assess whether there was any effect of age or body weight on CVC_D_ or CVC_D_/Ao_D_.

**Materials and methods:** We performed a single observer prospective observational study in standing healthy calves. BBCs were deemed healthy based on history and clinical evaluation. CVC_D_ and Ao_D_ were recorded by ultrasonography in a longitudinal plane at the PV and SX view.

**Results:** Seventeen BBC were enrolled, 10 male and 7 female, with a median age of 36 days (range 32-41 days) and median weight of 55 kg (range 46–79 kg). The CVC_D_ and Ao_D_ were obtained in all 17 BBC at the PV view, yet only in 2 at the SX view. Means were therefore not calculated for the SX view. The mean CVC_D_ was 0.94 cm (± SD = 0.13), the mean Ao_D_ was 1.11 (± SD = 0.11), with CVC_D_/Ao_D_ at 0.84 cm (± SD = 0.09). Statistical analysis failed to detect a direct correlation between age and weight with CVC_D_ (*P*_value_= 0.2 and 0.5, respectively) or CVC_D_/Ao_D_ (*P*_value_= 0.25 and 0.55, respectively).

**Conclusions:** CVC_D_ and Ao_D_ assessment was easily performed at the PV view, yet only rarely achieved at the SX view in awake and standing healthy calves. This pilot study failed to establish a direct correlation between age or weight and CVC_D_.

#### Comparison of thoracic point-of-care ultrasound and radiographic findings as well as clinical evolution and C-reactive protein concentrations in dogs treated for aspiration pneumonia

##### N. Rodrigues^1^, L. Giraud^1^, F. Billen^1^, C. Clercx^1^, S. Boysen^2^, K. Gommeren^1^

###### ^1^University of Liège, Faculty of Veterinary Medicine, Liège, Belgium; ^2^University of Calgary, Faculty of Veterinary Medicine, Calgary, Canada

####### **Correspondence:** Kris Gommeren, e-mail: krisjeg@hotmail.com

**Background:** Aspiration pneumonia (AP) is commonly diagnosed on history, clinical signs and thoracic radiography (RX). C-reactive protein (CRP) is used for diagnosis and follow-up of inflammatory conditions in veterinary medicine. Lung ultrasound (LUS) is described for diagnosis and follow-up of AP in human medicine. LUS findings in human and canine pneumonia include increased numbers of B-lines, lung consolidation and pleural effusion. The clinical evolution, CRP concentrations and changes identified on LUS and RX in canine AP have not been described or compared serially. This observational study was undertaken in the hopes of better guiding treatment decisions.

**Materials and methods:** Dogs with suspected AP based on compatible history and clinical signs, increased CRP and RX changes at presentation were prospectively recruited. LUS was performed at presentation (T0), after 2 weeks (T1) and 1 month (T2). At each time point clinical signs, CRP, RX and LUS findings were recorded. LUS was performed using a modified Armenise technique assessing 9 windows on each hemithorax, and the subxiphoid view. LUS lesions were considered mild (> 3 B-lines or coalescent B-lines), or severe (lung consolidation or pleural effusion) and their distribution was recorded. Lateral RXs were divided into 9 regions, arbitrarily corresponding to LUS windows, to compare approximative lesion distribution.

**Results:** Seventeen dogs were enrolled at T0, 13 were seen at a control visit at T1 of which 6 were also seen at T2. All dogs seen at T1 and T2 had complete resolution of clinical signs. CRP was increased (median 129 mg/dL) at T0, remained slightly increased in 2/13 dogs at T1 (14 and 32 mg/dL) and was normal in 6/6 dogs at T2. Radiographic lesions resolved in 2/13 and 4/6 dogs at T1 and T2, respectively. A shred sign was observed in 16/17 dogs, whereas tissue like signs and pleural effusion were not identified. 1/13 dogs showed a persistent but improving shred sign at T1 which was also noted at T2. Mild LUS lesions were still present in 8/13 and 4/6 dogs at T1 and T2, respectively. All patients had rapid resolution of clinical signs, whereas severe LUS lesions, increased CRP and radiographic lesions were still present in 1/13, 2/13 and 11/13 dogs at T1, respectively. Severe LUS lesions had comparable distributions to RX lesions. There was no correlation between mild LUS lesions and RX in 50% of cases.

**Conclusions:** Severe LUS lesions and CRP concentrations may correlate better with clinical findings than RX during serial evaluation of canine AP.

#### Evaluation of point-of-care ultrasound performed by non-cardiologists for subjective assessment of cardiac chambers size in dogs presented to the cardiology service

##### L. Giraud, K. Gommeren, A.-C. Merveille

###### University of Liège, Faculty of Veterinary Medicine, Liège, Belgium

####### **Correspondence:** Kris Gommeren, e-mail: krisjeg@hotmail.com

**Background:** Cardiac point-of-care ultrasound (POCUS) is a rapid and non-invasive method to screen for gross cardiac pathology. In human medicine, cardiac POCUS is widely used to assess cardiac chambers size and volume status, helping to guide diagnosis and treatment in unstable patients [1, 2]. Similarly, POCUS assessment of left atrial enlargement allows detection of left-sided congestive heart failure in companion animals [3, 4]. Combined with physical examination, it improves the detection of feline occult heart disease [5]. This study aimed to evaluate the agreement between POCUS performed by trained clinicians and echocardiography by a board certified cardiologist to assess cardiac chamber size in dogs presented to a cardiology service.

**Materials and methods:** Descriptive study. Two clinicians received a 2-h theoretical and 6-h practical course in cardiac POCUS prior to the study (right parasternal short and long axis views in right lateral recumbency). Dogs presented to the cardiology service between March 2019 and January 2020, were enrolled prospectively. The clinicians were informed about the dogs’ presenting clinical signs, but were blinded to the underlying structural heart disease and current medication. They subsequently performed a physical examination and cardiac POCUS on each dog. Left atrial (LA), left ventricular (LV), and right heart (RH) size were scored subjectively to be small, normal or enlarged. RH was evaluated as a whole because it is harder to assess than LH, and significant disease will likely affect both cavities. These subjective assessments were compared to echocardiographic results, performed by the cardiologist.

**Results:** Fifty-one dogs were included: 5 with physiological murmurs and no structural heart disease, 46 with heart disease. Degenerative mitral valve disease was diagnosed in 60% (31/51). POCUS subjective scores agreed with quantitative echocardiographic LA assessment in 98% (50/51), and with LV assessment in 84.3% (43/51) of cases. 52.9% (27/51) of dogs had LV enlargement, which POCUS correctly identified in 85.1% (23/27). LV size was overestimated in 12.5% (3/24) cases with no evidence of LV enlargement on echocardiography. RH scores were accurately assessed in 88.2% (45/51) of cases. However, only 9 displayed echocardiographic right cardiac enlargement, which POCUS correctly identified in only 44.4% (4/9).

**Conclusions:** Cardiac POCUS for subjective assessment of LA and LV size by clinicians having received an 8-h training has good agreement with echocardiography results performed by a cardiologist. Caution seems warranted for the assessment for RH size, although this population’s low incidence of right heart diseases prevents drawing strong conclusions.

**References:**

1. Labovitz AJ, Noble VE, Bierig M, et al. Focused cardiac ultrasound in the emergent setting: a consensus statement of the American Society of Echocardiography and American College of Emergency Physicians. *J Am Soc Echocardiogr.* 2010;23(12):1225-1230.
2. Spencer KT. Focused cardiac ultrasound: where do we stand? *Curr Cardiol Rep.* 2015;17(3):567.
3. Tse YC, Rush JE, Cunningham SM, Bulmer BJ, Freeman LM, Rozanski EA. Evaluation of a training course in focused echocardiography for noncardiology house officers. *J Vet Emerg Crit Care (San Antonio).* 2013;23(3):268–273.
4. Ward JL, Lisciandro GR, Ware WA, et al. Evaluation of point-of-care thoracic ultrasound and NT-proBNP for the diagnosis of congestive heart failure in cats with respiratory distress. *J Vet Intern Med.* 2018;32(5):1530–1540.
5. Loughran KA, Rush JE, Rozanski EA, Oyama MA, Larouche-Lebel É, Kraus MS. The use of focused cardiac ultrasound to screen for occult heart disease in asymptomatic cats. *J Vet Intern Med.* 2019;33(5):1892–1901.

#### The use of veterinary point-of-care ultrasound by veterinarians: a nationwide Canadian survey

##### J. Pelchat, S. Chalhoub, S. Boysen

###### Department of Veterinary Clinical and Diagnostic Sciences, Faculty of Veterinary Medicine, University of Calgary, Canada

####### **Correspondence:** Jen Pelchat, e-mail: jip756@mail.usask.ca

**Background:** Veterinary point-of-care ultrasound (VPOCUS) is frequently employed as a diagnostic, monitoring, and triage tool in companion animals, and is being integrated into many student veterinary curriculums. Lack of standardization and training creates conflicting views on what VPOCUS should include, particularly regarding abdominal and thoracic focused assessment with sonography for trauma (AFAST/TFAST). The objective of this study was to assess VPOCUS protocols across Canada through a nationwide survey.

**Materials and methods:** An anonymous Survey Monkey VPOCUS questionnaire was distributed nationwide via electronic newsletters by the Canadian Veterinary Medical Association and various provincial veterinary medical associations.

**Results**: Seventy-eight veterinarians from across Canada responded to the survey, with a 92% survey completion rate (*n* = 74). Among respondents, 88% perform ultrasound. Reasons for not performing VPOCUS (in general, AFAST or TFAST specific) included lack of a machine (up to 50%), lack of experience and confidence in identifying and diagnosing pathology (up to 40%), and lack of ultrasound training or education (up to 30%). Of those who perform ultrasound, 94% perform AFAST and 69% TFAST. The majority of AFAST scans include the 4 standard sites, only 28% scan the umbilical site. Respondents felt confident diagnosing peritoneal free fluid (98%), pyometra (80%), and urinary bladder wall thickness and urine volume estimation (66% and 63%, respectively). Pathology diagnosed with less confidence include gall bladder halo signs (40%), retroperitoneal injury (18%) and pneumoperitoneum (18%). For AFAST respondents, 67% perform serial scans but few document abdominal fluid scores (40%). During TFAST the pericardial site was assessed by 97%, and the chest tube and subxiphoid sites by approximately 50% of respondents. 93% and 100% of respondents are confident assessing pleural and pericardial effusion, respectively. There was less confidence identifying the glide sign (56%), B-lines (57%), left atrium to aorta ratio (22%), subjective cardiac contractility (27%), vascular volume estimation (vena cava diameter) (4%), and subpleural consolidation (4%). Only 22% include regional lung ultrasound, most often searching for B-lines (76%), with few assessing lung surface irregularities (40%), air bronchograms (9%), pulmonary thromboembolisms (9%), lung nodules (38%), tissue signs/hepatization (29%), or shred sign (11%).

**Conclusions:** Across Canada, the majority of respondents perform VPOCUS, with AFAST performed more frequently and confidently than TFAST, regional lung and cardiovascular ultrasound. AFAST scans are more standardized than TFAST scans. More training, education, and standardization of techniques appear to be key elements to help build confidence and experience, particularly regarding TFAST applications and diagnosis.

#### Comparison of text-versus-video teaching strategies for pleural and lung ultrasound learning in a veterinary student population

##### S. Wells, A. Romero, C. Wagg, J.-Y. Tan, R. Archer, S. Boysen

###### University of Calgary, Faculty of Veterinary Medicine, Calgary, Canada

####### **Correspondence:** Søren Boysen, e-mail: srboysen@ucalgary.ca

**Background:** There are no studies comparing written text to instructional video as an ultrasound learning strategy. Veterinary clinicians may rely on written text, instructional video, or both if they cannot attend hands-on training due to cost, time, and/or travel. We hypothesize there will be a difference in technical skill and confidence between written text and instructional videos for veterinary students learning pleural space and lung (PLUS) ultrasound techniques.

**Materials and methods:** Randomized prospective trial. Human and animal ethics was obtained (AC17-0069, REB17-0889). A teaching video and written text containing identical background information and procedural detail was created. Forty year 1–3 veterinary students (*n* = 12, 16, 12, respectively) were randomly assigned to review a written manual or instructional video for 20 min. An Objective Structured Clinical Examination was used to assess each student’s PLUS knowledge and technique on a cadaver prior to accessing the instructional material, and on 4 cadavers after viewing the instructional material. A positive pressure ventilation cadaver model was used. Students were blinded to PLUS pathology: pneumothorax, pleural effusion, single to multiple B, or no pathology. Each student had 7 min to complete each station. Linear regression analysis with repeated measures determined if students using either format demonstrated improvement in overall score and level of PLUS completion. A pre- and post-procedural survey was used to assess student PLUS confidence.

**Results:** Year 3 students scored higher than Year 1 students through all stations. The video group scored higher for identification and quantification of B-lines, regardless of group year. There were no differences in the diagnosis of pleural effusion or pneumothorax. Pre- and post-examination surveys revealed increased confidence in performing ultrasound and identifying pathology across all groups with no differences in perceived confidence between teaching formats. There were significantly greater scores for all students between Stations 1 and 2 and between Stations 2 and 5.

**Conclusions:** There is little difference in learning and confidence in performing PLUS when comparing video to written text, with the exception of B-line identification. B-lines are dynamic in nature (they must move to be accurately identified), which may explain why video learning appeared better than text for this PLUS skill. Both written text and instructional video teaching modalities, along with repeated performance, demonstrate improved learner performance and confidence, however, further studies are required to determine the recommended number of scans required for veterinary students to become competent in performing PLUS scans.

#### Veterinary point-of-care ultrasound probe orientation for detection of pleural effusion in dog cadavers by novice sonographers: a pilot study

##### S. Boysen, S. Chalhoub, A. Romero

###### University of Calgary, Faculty of Veterinary Medicine, Calgary, Canada

####### **Correspondence:** Serge Chalhoub, e-mail: schalhou@ucalgary.ca

**Background:** Evidence suggests probe orientation and location affect the sensitivity of veterinary point-of-care ultrasound (VPOCUS) to detect pleural effusion. The objective of this study was to compare two VPOCUS protocols performed by novices for identification of pleural effusion in positive pressure ventilated (PPV) canine cadavers. We hypothesize there will be a difference between protocols for identification of pleural effusion.

**Materials and methods:** Ethics approval was obtained (AC17-0092, REB19-1405). Eight interns (< 10 h ultrasound experience) received 90 min of pleural space and lung pathology lecture and 1 h of TFAST, Vet-BLUE and PLUS (recently published) training on live dogs. Four thawed cadavers (25–30 kg), placed in sternal recumbency, were scanned for pleural effusion; water was added to the pleural space when required to create large, scant, moderate, and small quantities of effusion, respectively, as subjectively and independently determined by two experienced sonographers. Interns were blinded to the presence or absence of pleural effusion. PPV cadavers were scanned bilaterally by interns, randomly starting with one of two protocols, alternating protocols for each subsequent cadaver. Protocol 1 (P1): probe perpendicular to the ribs, starting at the costochondral junction, sliding cranially and caudally similar to Vet-BLUE (ventral and dorsal movement allowed). Protocol 2 (P2): probe parallel to the ribs, starting at the costochondral junction, sliding ventrally to find the ventral pleural space and lung borders, sweeping cranially from the diaphragm to the thoracic inlet as per PLUS (ventral and dorsal movement allowed). Interns were timed and recorded pleural fluid as present/absent. Results were analyzed using McNemar’s test. Due to small sample size, scant-to-small fluid groups, and moderate-to-large groups were combined for statistical analysis. *P* ≤ 0.05 was considered significant.

**Results:** Each participant scanned 8 hemi-thoraces. Participants completed all protocols in < 5 min. Overall there was no significant difference between protocols for detection of pleural effusion (P1 *n* = 16/32, P2 *n* = 27/32). There was no statistical difference for moderate/large volume pleural effusion (P1 *n* = 14/16, P2 *n* = 14/16), but there was for scant/small volume pleural effusion (P1 *n* = 5/16, P2 *n* = 13/16, respectively, *P* = 0.048).

**Conclusions:** The accuracy of VPOCUS protocols for detection of pleural effusion by novices in PPV cadavers varies, particularly when fluid quantity is scant/small. This is likely explained by protocol differences in probe orientation and location. Further research is required to validate the accuracy of VPOCUS probe orientation and location for detection of pleural effusion in spontaneously breathing companion animals.

